# Supplementary figures and images for: Vaginal and urinary evaluation of lactobacilli quantification by qPCR: Identifying factors that influence urinary detection and the quantity of Lactobacillus
Source: PLoS One. 2023 Apr 14;18(4):e0283215. doi: 10.1371/journal.pone.0283215 (PMC10104322; doi:10.1371/journal.pone.0283215)

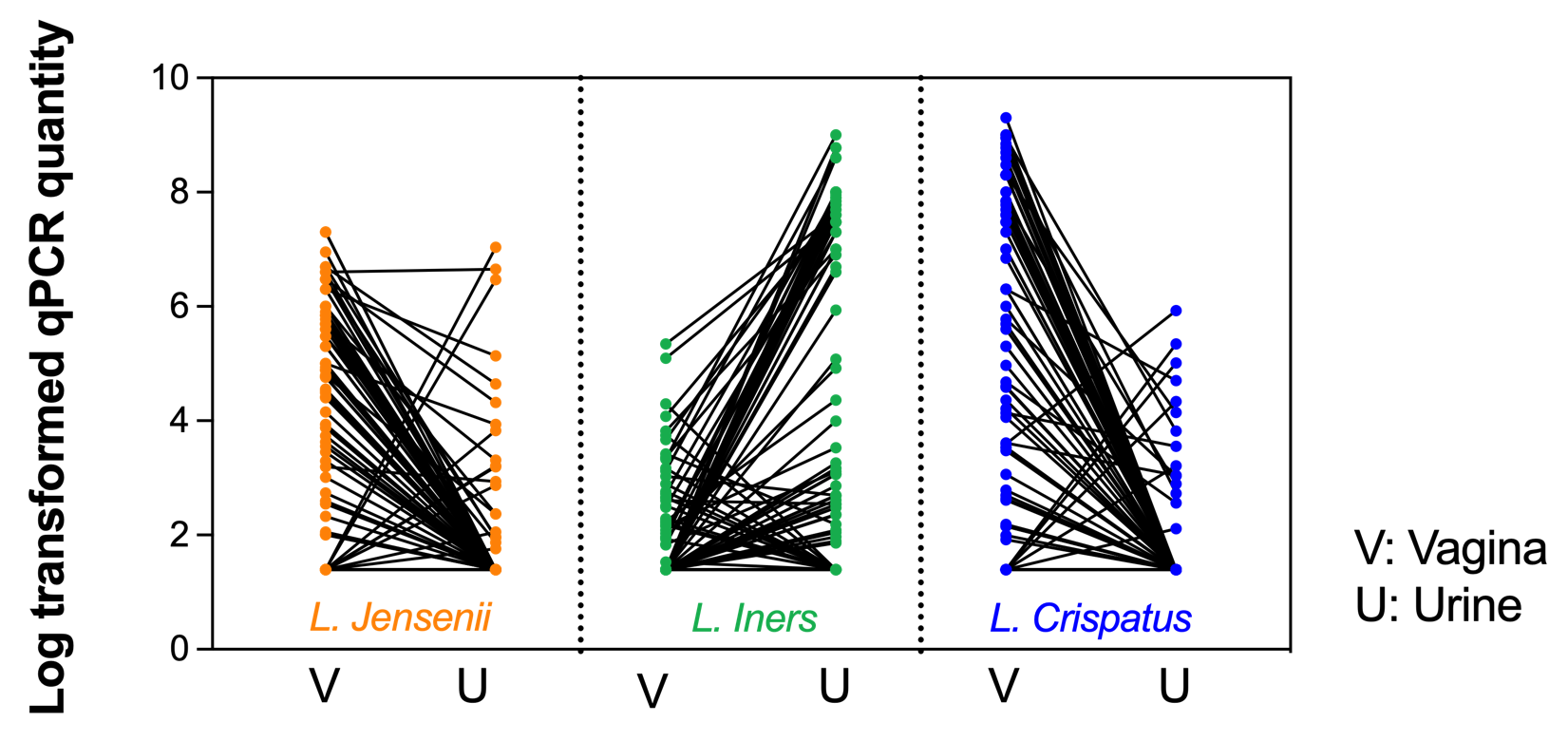

Supplement: S1 Fig — This figure includes samples without detectable Lactobacillus species in the urine sample. (TIF) [file pone.0283215.s001.tif]
