# Supplementary material for: Vaginal and urinary evaluation of lactobacilli quantification by qPCR: Identifying factors that influence urinary detection and the quantity of Lactobacillus
Source: PLoS One. 2023 Apr 14;18(4):e0283215. doi: 10.1371/journal.pone.0283215 (PMC10104322; doi:10.1371/journal.pone.0283215)
Supplement: S1 Table — (DOCX) [file pone.0283215.s002.docx]

| PCR assay | PCR conditions | Amplicon size | Primer/Probe | Primer/Probe Sequence | References |
| --- | --- | --- | --- | --- | --- |
| *Lactobacillus iners* | 55°C annealing, 39 s | 76 bp | 165F_Liners | 5’-GATGCTAATACCGGATAAYAACAGAT-3’ | [1] |
|  | 72°C extensions, 30 s |  | 241R_Liners | 5’-CACCGCAGGTCCATCCAAGA-3’ |  |
| *Lactobacillus jensenii* | 61°C annealing, 30 s | 69 bp | 988_Ljens | 5’-GTCTTGACATCCTTTGACCAC-3’ | [1] |
|  | 72°C extension, 30 s |  | 1057R_Ljens | 5’-CATGCACCACCTGTCTCTTT-3’ |  |
| *Lactobacillus crispatus* | 63°C annealing, 39 s | 66 bp | 989F Lcrisp | 5’-TCTTGACATCTAGTGCCATTTGT-3’ | [2] |
|  | 72°C extension, 30 s |  | 1055R Lcrisp | 5’-TGCACCACCTGTCTTAGC-3’ |  |

**S1 Table. Primers, probe sequences, and PCR conditions for TaqMan assays**
