# Supplementary material for: Vaginal and urinary evaluation of lactobacilli quantification by qPCR: Identifying factors that influence urinary detection and the quantity of Lactobacillus
Source: PLoS One. 2023 Apr 14;18(4):e0283215. doi: 10.1371/journal.pone.0283215 (PMC10104322; doi:10.1371/journal.pone.0283215)
Supplement: S2 Table — (DOCX) [file pone.0283215.s003.docx]

**S2 Table. Association between vaginal and urinary 16S rRNA gene copy numbers of *L. jensenii, L iners* and *L. crispatus,* excluding samples without quantifiable detection in the urine.**

|  | Regression coefficient^a^ | 95% CI | P value |
| --- | --- | --- | --- |
| *L. jensenii* | 0.16 | 0.023-0.43 | 0.032 |
| *L. iners* | 0.031 | -0.43-0.50 | 0.88 |
| *L. crispatus* | -0.24 | -0.53-0.043 | 0.088 |
| ^a^For linear regression associating vaginal with urinary 16rRNA gene copies/sample for each species, adjusted for Nugent score (values log_10_ transformed for analysis) | | | |
