# Supplementary material for: Vaginal and urinary evaluation of lactobacilli quantification by qPCR: Identifying factors that influence urinary detection and the quantity of Lactobacillus
Source: PLoS One. 2023 Apr 14;18(4):e0283215. doi: 10.1371/journal.pone.0283215 (PMC10104322; doi:10.1371/journal.pone.0283215)
Supplement: S3 Table — (DOCX) [file pone.0283215.s004.docx]

**S3Table. Association between vaginal and urinary detection of the same *Lactobacillus* species (N=93)**

|  | Number of urinary samples with detectable *Lactobacillus* species | | |
| --- | --- | --- | --- |
| Number of vaginal samples with the same detectable *Lactobacillus* species | ***L. jensenii*** |  |  |
|  | Detected | Undetected | p-value |
| Detected | 29 | 29 |  |
| Undetected | 5 | 30 | 0.001 |
|  | ***L iners*** |  |  |
| Detected | 15 | 49 |  |
| Undetected | 5 | 24 | 0.50 |
|  | ***L. crispatus*** |  |  |
| Detected | 11 | 50 |  |
| Undetected | 4 | 28 | 0.49 |
