# Supplementary material for: Vaginal and urinary evaluation of lactobacilli quantification by qPCR: Identifying factors that influence urinary detection and the quantity of Lactobacillus
Source: PLoS One. 2023 Apr 14;18(4):e0283215. doi: 10.1371/journal.pone.0283215 (PMC10104322; doi:10.1371/journal.pone.0283215)
Supplement: Attachment — Submitted filename: Reviewer-Manuscript ID-1033431.pdf [file pone.0283215.s005.pdf]

Reviewer Report– Manuscript Number PONE-D-22-27069

Congratulations to the authors for a beautiful study and I believe that the present study should be published!

Said that, please find my general and minor comments below.

General comments

I recommended the present study for publication. However, the manuscript could be improved by the authors, and I made some suggestions in my report such as:

It is well-known that a midstream clean catch urine sample does not guarantee a sample without bacterial contamination from the penis or vagina from getting into a urine sample. This type of sample collection represents a main limitation of the present study; however, the authors recognized it at the end of the *Discussion* section of the study. Nonetheless, the number of women was also very low, and it should be mentioned in the limitations of the study.

The references and introduction could be improved (see my minor comments).

A better description of the *Material and Methods* section with a more exhaust explanation of the experimental design of this study must be realized by the authors.

The *Discussion* section of the manuscript needs to be improved by the authors, discussing better the detected lactobacilli species by themselves and as consortia in the vaginal microbiota and their potential role in the bladder microbiota/urinary microbiome (see my minor comments).

Minor comments

Abstract

Line 33- Please put “L. crispatus” in italic form.

Introduction

Lines 87-89- I invite the authors to clarify what type of significant concordance was found by DNA sequencing between vaginal and urine samples in the cited studies. This further information would be useful to the Readers. Likewise, more similar studies of urine samples could be added to obtain a better scenario for the Readers.

Materials and Methods

Lines 101-103- Plea rewrite the exclusion criteria sentence, more exactly: “Women were excluded if their age was under 21 years if they were pregnant, had known HIV infection or immunosuppression due to medication, or were prisoners.”

Lines 108-109- It is well-known that a midstream clean catch urine sample does not guarantee a sample without bacterial contamination from the penis or vagina from getting into a urine sample. This type of sample collection represents a main limitation of the present study; however, the authors recognized it at the end of the *Discussion* section of the study.

Line 118, 128, and 129- Please replace “ul” and “uL” with “µL”. Please check this type of error in the remaining manuscript.

Line 130- “Quantity was calculated using a plasmid standard curve”. Please briefly describe the standard curve for the Readers instead of just mentioning that it was previously described (what I think the authors are referring to the reference 17 in the previous sentence).

Lines 133-136- Please state the controls used in the qPCR assays. There is no mention or description of controls in qPCR assays, which is concerning for this reviewer.

Supplemental table 1- Please rectify the lack of units in some protocols of the “PCR conditions” column.

Results

Figure 1 does not add any useful information to the Readers. Better to add supplemental table 3 as a main table in the results.

In table 1- Please put “spp” in a non-italic form. Also, the “Not detected” column stated N= 44 but, in the race section, the authors only described 43 patients (39, 3, and 1).

Other mistakes were found such as different p-values, *P* values, and *p* values in main or supplemental tables or wrongly lactobacilli names (such as *L. Jensenii* and *L. Crispatus* in Supplemental table 3 and even in Figure 2 plus Supplemental Figure 1. So, I strongly recommend that the authors revised the tables/figures of the entire *Results* section.

Figure 2 and Supplemental Figure – The resolution is bad, and no quantitative values are clear to this reviewer. I suggest the authors amend the figures or figure out another way to illustrate these quantitative data.

Line 199- “…even after adjusting for the Nugent score (Table 2).” Please clarify how did you adjust the Nugent score.

Table 3- Please clarify how the authors evaluated the false discovery rate (FDR) in the multivariate logistic regression analysis for urinary detection of *L. jensenii*, *L. iners*, and *L. crispatus*. The evaluation of FDR in the multivariate logistic regression is not clear to me. Also, please put vaginal in the non-italic form at the “*Vaginal Lactobacillus*” line.

Discussion
Lines 243 and 270- Again, please put “spp.” In a non-italic form.

A better discussion is needed. Two pages are not enough to discuss the obtained results. The author generally discussed the *Lactobacillus* genus and briefly mentioned the role of *L. gasseri*. Also, the authors stated in lines 265-266 “*L. crispatus* has been well established as an essential organism in urinary health (3,8,11,12,20).” How *L. crispatus* is well established as an essential microorganism in the urinary tract? Explain to the Readers… because *L. crispatus* is well-known probiotic species in the vaginal tract preventing the colonization of opportunistic or primary pathogens and consequently urinary infections as well. The authors must discuss better his role in the urinary tract.

Also, probiotic activity is caused not only by individual *Lactobacillus* species but also by its multi-microbial interaction. Please read the recent publication (https://www.frontiersin.org/articles/10.3389/fcimb.2022.863208/full) and improved the discussion about *L. iners, L. crispatus,* and *L. jensenii* role in the vaginal microbiota and their potential role as part of the bladder microbiota/urinary microbiome.

In addition, the number of women was also very low, and it should be mentioned in the limitations of the study.

Congratulations on the present study. However, the manuscript needs obligatorily to be improved in order to be published. The *Discussion* and *Material and Methods* sections of the manuscript need to be improved by the authors.
